# Vultures of the Seas: Hyperacidic Stomachs in Wandering Albatrosses as an Adaptation to Dispersed Food Resources, including Fishery Wastes

**DOI:** 10.1371/journal.pone.0037834

**Published:** 2012-06-06

**Authors:** David Grémillet, Aurélien Prudor, Yvon le Maho, Henri Weimerskirch

**Affiliations:** 1 CEFE-CNRS, UMR5175, Montpellier, France; 2 Percy FitzPatrick Institute and DST-NRF Centre of Excellence at the University of Cape Town, Rondebosch, South Africa; 3 CEBC-CNRS, UPR1934, Villiers en bois, France; 4 Institut Pluridisciplinaire Hubert Curien, Université de Strasbourg and CNRS UMR7178, Strasbourg, France; Institute of Ecology, Germany

## Abstract

Animals are primarily limited by their capacity to acquire food, yet digestive performance also conditions energy acquisition, and ultimately fitness. Optimal foraging theory predicts that organisms feeding on patchy resources should maximize their food loads within each patch, and should digest these loads quickly to minimize travelling costs between food patches. We tested the prediction of high digestive performance in wandering albatrosses, which can ingest prey of up to 3 kg, and feed on highly dispersed food resources across the southern ocean. GPS-tracking of 40 wandering albatrosses from the Crozet archipelago during the incubation phase confirmed foraging movements of between 475–4705 km, which give birds access to a variety of prey, including fishery wastes. Moreover, using miniaturized, autonomous data recorders placed in the stomach of three birds, we performed the first-ever measurements of gastric pH and temperature in procellariformes. These revealed surprisingly low pH levels (average 1.50±0.13), markedly lower than in other seabirds, and comparable to those of vultures feeding on carrion. Such low stomach pH gives wandering albatrosses a strategic advantage since it allows them a rapid chemical breakdown of ingested food and therefore a rapid digestion. This is useful for feeding on patchy, natural prey, but also on fishery wastes, which might be an important additional food resource for wandering albatrosses.

## Introduction

The capacity of animals to survive and reproduce in a given environment is often seen as primarily limited by energy acquisition (the metabolic theory of ecology [1]). Yet two additional bottlenecks occur: (a) their ability to shed excess heat generated by muscle activity (heat dissipation limit theory [2]), and (b) their capacity to digest food. This latter alternative has long been neglected, yet Karasov, Diamond and colleagues demonstrated the existence of digestive bottlenecks in a series of species, hummingbirds (e.g. *Selasphorus rufus*) being the classic example [3], [4]. Ecologically, digestion is a fundamental process since it does not only condition the fate of individual organisms, but also the flow of matter and energy across food webs [5].

Biologically, digestion serves the purpose of breaking down and assimilating ingested food. In the digestive tract it is aided by mechanical churning, low pH, digestive enzymes, and the occasional symbiont [6]. The severity of this process largely depends upon the texture and hardiness of the food: when the aforementioned hummingbird feeds, nectar is easy to break down. At the other extreme, ostrich (*Struthio struthio*) food is proverbially tough.

In particular, generalists and/or scavengers need to be able to digest a broad diet, including hardy food [7]. Moreover, foraging theory predicts that animals feeding on patchy food should be capable of ingesting large amounts, and to digest them as quickly as possible [8]. This is particularly marked in birds which need to become airborne, even after the largest meals. A prime example of this strategy is found in vultures feeding on carrion. These species have large stomachs, and also very low stomach pH (1.5) which plays a crucial role in chemically dissolving hard parts, especially bones [9]. A pH of 1 to 2 is also optimal for proteolytic enzymes that play a crucial role in the breakdown of food [10].

In the Southern Ocean, series of studies have addressed the capacity of marine predators to acquire food [11], but little is known about their digestive physiology and potential digestive bottlenecks. In seabirds, pioneering work demonstrated that some prey, in particular squid, are more difficult to digest than others, that feeding on squid leads to delayed gastric emptying [12], and that birds eating squid tend to have longer digestive tracts [13].

Wandering albatrosses (*Diomedea exulans*), the largest extant seabird species, primarily feed on squid caught at the ocean’s surface [14]. However their diet is not restricted to squid, but shows a large variety of other prey such as fishes, carrion of seabirds and marine mammals, as well as fishery wastes, whose proportion vary according to sites or stages of the breeding season [15]–[18]. Wandering albatross food occurs in discrete and unpredictable patches; birds fly for extended periods before ingesting large squid or other prey at irregular intervals [19]. The most profitable predatory strategy is therefore to ingest as much food as possible whenever available and to move to another patch [20]. Albatross stomach morphology reflects this evolutionary constraint, with an estimated volume of 3–4 L [21], which allows birds to ingest large single prey items of up to 3.2 kg [19], i.e. over 30% of their own body mass. After such large meals, wandering albatrosses may have difficulties to take off if wind conditions are not favourable, which explains why they often remain at the ocean surface for several hours [22]. If they do manage to take off rapidly (in strong winds), such additional food load may increase their flight costs by increasing wing loading [23]. Wandering albatrosses therefore clearly should process large meals as quickly as possible, a strategy that they theoretically share with vultures that face similar foraging and flight constraints.

In this context, we tested the hypothesis that wandering albatrosses are vultures of the seas, designed to rapidly digest large volumes of hardy food such as squid, and are therefore pre-adapted to rapidly process fishery waste, a recently occurring resource that provides large quantities of food during a short period of time. To address this issue, we performed GPS-tracking of wandering albatrosses at sea, and recorded their stomach pH during, and in-between meals. These pH levels were then compared with those of other seabird species feeding on a variety of food types and with vulture stomach pH to test the prediction that wandering albatross stomach pH is as low as that of vultures.

## Methods

### Ethics Statement

All scientific procedures were validated by the ethics committee of the French Polar Institute (IPEV), were conducted according to its guidelines and under permits of the Réserve Naturelle des Terres Australes and of the Comité de l’Environnement Polaire.

The study was conducted in January – March 2011 on Possession Island (46°S, 51°E), Crozet Archipelago, Southern Ocean. Wandering albatrosses were studied while incubating, a period during which parents take shifts at the nest while a partner forages at sea for periods of a few days to a month [24]. Birds were caught at the nest within the framework of a long-term monitoring program of their foraging behaviour. Great care was taken to minimize stress while handling, which lasted <10 min in all cases. Birds were either fitted with a GPS data logger to record their movements at sea, or with a pH data logger to record stomach pH.

### GPS Positioning

We used miniaturized GPS recorders (i-gotU, Mobile Action Technology Inc, New Taipei City, Taiwan; 44.5×28.5×13 mm, 20 g i.e. 0.2% bird body mass) attached with waterproof tape to feathers. Birds were captured and fitted with the GPS after they have been relieved by their partner and were about to leave for a foraging trip at sea. Device and tape were removed upon return to the colony after a single foraging trip. This technique has been successfully used on this species for nearly two decades [25], with no measurable effects on behaviour, reproductive output or survival [26]. Devices were programmed to record a GPS position every 15 min across the foraging trip. Stored data were mapped on Google Earth® to illustrate wandering albatross at-sea home range.

### Stomach pH and Temperature Recordings

We studied stomach pH and temperature using autonomous, miniaturized recorders enclosed in a titanium housing that was swallowed by the birds and remained in the stomach for the time of the measurement. The devices used (pH-meter, Earth & Ocean Technologies, Kiel, Germany, 11 cm long, 2 cm in diameter, mass 80 g i.e. 0.9% of bird body mass) are fully described in [27], which also provide all necessary details about preparation, calibration procedures and data handling. Devices were set to record pH (accuracy 0.02 pH units) and temperature (accuracy <0.1°C) every ten seconds. Temperature data were analysed following [21] and [28] so as to estimate the mass of prey caught at sea using the amplitude and the duration of the temperature drop recorded in the stomach after prey ingestion.

The deployment procedure in the field closely followed previous investigations conducted in the same species [28], using devices of the same mass and size, which nonetheless only recorded stomach temperature: Birds were induced to swallow the pH-meter at the beginning of the experiment, and it was recovered at the end of the measurement by stomach flushing, a technique which has been routinely used to gather stomach contents of seabirds for the purpose of dietary studies [29].

## Results

### GPS-tracking

**Figure 1:**
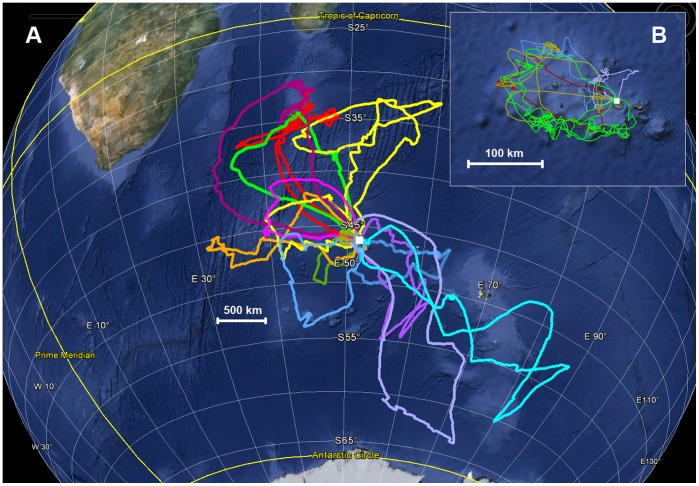
Foraging paths of 40 incubating wandering albatrosses from Possession Island, Crozet archipelago (white square) in January – March 2011. (A). Five birds performed long trips towards northwest, three performed long trips towards southeast, five birds performed intermediate trips, nine birds remained between the Crozet Archipelago and the westward Prince Edward Islands, and 18 birds remained on the Crozet plateau (B), extensively foraging along its edge; suggesting local interactions with fishing vessels.

We equipped a total of 43 birds with GPS recorders. One device did not collect data, a second was lost at sea, and a third only collected data for 12 hours. Therefore a total of 40 complete tracks were collected, for at-sea journeys of between 3.6 and 21.1 days (mean 9.3±4.9), during which birds travelled between 475 and 4507 km (mean 3511±2718). As demonstrated in previous work, the duration of trips was very variable, with trips occurring over oceanic waters, as well as over the shelf edge (Fig. 1).

### Stomach Temperature and pH Patterns

We equipped a total of 5 birds with pH-meters. Two individuals were equipped for a few hours at the nest during an initial test phase, while three were equipped before going out to sea. Within the latter group, only one bird came back to the nest with its pH-meter, the two others regurgitated the device at sea, something which had already occurred in previous studies using similar stomach loggers [28], as it is the natural mechanisms by which wandering albatrosses and other seabirds evacuate indigestible food parts, such as squid beaks.

**Figure 2:**
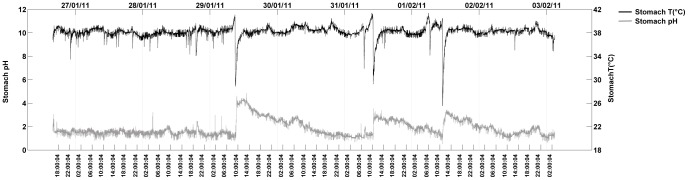
Parallel recordings of stomach temperature (upper trace, right scale) and stomach pH (lower trace, left scale) in a free-ranging wandering albatross during a seven-day foraging trip at sea.

We therefore analyzed stomach pH and temperature recordings for three birds. In the bird that went out to sea (for a period of 7 days, Fig. 2), basal stomach pH was extremely low (pH 1.35±0.14), occasionally decreasing to pH 0.51. Parallel temperature recordings indicated ingestion of cold prey (Fig. 2), who’s estimated mass was on average 110±280 g. Prey items were occasionally large, up to an estimated 1160 g. After the intake of such large items, stomach pH rose sharply (up to pH 4.88), and re-acidification to baseline levels only occurred within several hours to one day (Fig. 2). The two birds that stayed on the nest and did not feed showed stable, very low stomach pH levels (average pH 1.50±0.13 and 1.65±0.10, respectively). These values are in line with the ground pH level recorded in the bird that went out to sea, and the average baseline pH was therefore pH 1.50±0.13 across all three birds.

## Discussion

**Figure 3:**
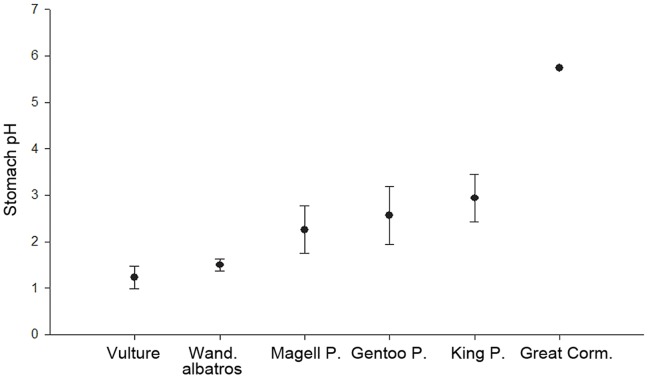
Baseline stomach pH (mean and standard deviation) in white-backed griffon vultures [30], wandering albatrosses (this study), Magellanic and gentoo penguins (*Spheniscus magellanicus* and *Pygoscelis papua*, [35]), king penguins (*Aptenodytes patagonicus*, [27]) and great cormorants (*Phalacrocorax carbo*, [36]).

Using the first stomach pH recording ever conducted in a foraging petrel, we validate our prediction that the stomach pH of wandering albatrosses is extremely low (Fig. 2). Such low pH is very close to the baseline stomach pH recorded in white-backed griffon vultures (Fig. 3, [30]), and is significantly lower than pH levels recorded in a variety of other seabird species that mainly feed on fish and were previously studied using the same miniaturised, autonomous pH-meters (Fig. 3).

Our findings are based upon a very limited sample size, consisting of only one recording at sea and two for birds at the nest. They should be complemented by further recordings on a larger number of birds across different stages of the breeding cycle and also across different petrel species showing contrasting dietary preferences. However, our three recordings show consistent, extremely low baseline pH levels of 1.5 on average. Such physiological parameters are unlikely to show strong inter-individual variability, and indeed standard deviations for stomach pH measurements conducted in other bird species are within the same pH unit. We are therefore confident that our recordings demonstrate highly acidic (<2) stomach pH in wandering albatrosses.

Such low pH favours rapid chemical digestion of the food and is also optimal for proteolytic enzyme kinetic [10]. It is likely that this physiological characteristic evolved as a response to a diet largely composed of squid, and to a patchy distribution of this food resource resulting in large, infrequent meals. The strategy of wandering albatrosses is indeed to cover long distances rapidly and at low costs, to increase the probability of encountering dispersed prey patches whose distribution is unpredictable [22], [31]. They catch on average one prey every 200 km, and some prey can be as heavy as 3.2 kg [22], an additional load that increases wing loading and reduce optimality of flight [23], [32]. As indicated above, they often remain at the sea surface for several hours after having swallowed large prey items [22]. This time spent on the sea surface without capturing additional prey probably corresponds to their digestion time, a period during which low stomach pH allows them to process food quickly, to become airborne again and fly at the lowest-possible energetic costs [31]. Being able to digest rapidly large meals represents an important advantage by reducing time spent on the water, or flight costs. This strategy is the marine equivalent to that of foraging vultures, which also remain on the ground after large meals.

However, low stomach pH represents also a strategic advantage for seabirds feeding upon fishery wastes: they can absorb large volumes of this patchy resource, and digest them rapidly. Direct observations around the Crozet-Kerguelen islands conducted from long-liners producing wastes (A. Prudor, unpubl data) show that wandering albatrosses are the dominant species within multi-species flocks attending fishing vessels because of their large body size and aggressive behaviour [31]. They also have sufficient stomach volume to ingest large volumes of these wastes, yet after a large meal they typically stay at the ocean’s surface for several hours.

Wandering albatrosses from the Crozet islands are thought to feed to some extent on wastes from long liners harvesting Patagonian toothfish (*Dissostichus eleginoides*), yet the amount of fishery waste that they actually consume remains to be determined, as well as the incidence of this artificial food resource upon seabird apparent fitness. Indeed, fishery wastes are generally beneficial to scavenging seabirds [33], yet in certain cases they set ecological traps and diminish reproductive success [34].
